# A Hybrid Neural Network–Particle Swarm Optimization Informed Spatial Interpolation Technique for Groundwater Quality Mapping in a Small Island Province of the Philippines

**DOI:** 10.3390/toxics9110273

**Published:** 2021-10-21

**Authors:** Kevin Lawrence M. De Jesus, Delia B. Senoro, Jennifer C. Dela Cruz, Eduardo B. Chan

**Affiliations:** 1 School of Graduate Studies, Mapua University, Manila 1002, Philippines; klmdejesus@mymail.mapua.edu.ph (K.L.M.D.J.); jcdelacruz@mapua.edu.ph (J.C.D.C.); 2 School of Chemical, Biological, Materials Engineering and Sciences, Mapua University, Manila 1002, Philippines; 3 Resiliency and Sustainable Development Center, Yuchengco Innovation Center, Mapua University, Manila 1002, Philippines; 4 School of Civil, Environmental and Geological Engineering, Mapua University, Manila 1002, Philippines; 5 School of Electrical, Electronics and Computer Engineering, Mapua University, Manila 1002, Philippines; 6 Dyson College of Arts and Science, Pace University, New York, NY 10038, USA; echan@pace.edu

**Keywords:** groundwater, acid mine drainage, heavy metals, physicochemical characteristics, neural network, particle swarm optimization, spatial interpolation

## Abstract

Water quality monitoring demands the use of spatial interpolation techniques due to on-ground challenges. The implementation of various spatial interpolation methods results in significant variations from the true spatial distribution of water quality in a specific location. The aim of this research is to improve mapping prediction capabilities of spatial interpolation algorithms by using a neural network with the particle swarm optimization (NN-PSO) technique. Hybrid interpolation approaches were evaluated and compared by cross-validation using mean absolute error (MAE) and Pearson’s correlation coefficient (R). The governing interpolation techniques for the physicochemical parameters of groundwater (GW) and heavy metal concentrations were the geostatistical approaches combined with NN-PSO. The best methods for physicochemical characteristics and heavy metal concentrations were observed to have the least MAE and R values, ranging from 1.7 to 4.3 times and 1.2 to 5.6 times higher than the interpolation technique without the NN-PSO for the dry and wet season, respectively. The hybrid interpolation methods exhibit an improved performance as compared to the non-hybrid methods. The application of NN-PSO technique to spatial interpolation methods was found to be a promising approach for improving the accuracy of spatial maps for GW quality.

## 1. Introduction

Acid mine drainage (AMD) is a natural or man-made environmental occurrence that transpires when sulfide minerals are exposed to weathering conditions or as a result of specific mining activities. It is a discharge with low pH, high heavy metal, and deadly component concentrations. It is also typically formed when sulfide-abundant wastes have been introduced to the environment. This condition has been viewed as a severe environmental issue encountered by mineral extraction enterprises around the world [1,2]. AMD is caused by oxidation of pyrite and other sulfate metals when mining sources are exposed to air, microbial activity, and water. Among the potentially hazardous dissolved metals found in high quantities, iron (II) is the most prevalent and frequent in the majority of AMD locations. Iron (II) in AMD interacts with dissolved oxygen to form iron oxide precipitates often referred to as yellow boy and may kill life all along river or stream banks [3,4]. This problem has been identified as a significant environmental concern for mineral extraction companies worldwide. AMD is prevalent in both active and inactive or abandoned mining sites. However, it is less prevalent in current producing mines owing to pumping that kept the water table low. AMD is severe in closed and abandoned mines when pumps are shut off, causing water tables to recover [5]. In Asia [6,7], Europe [8], South America [9], North America [10], Africa [11], and Oceania [12], AMD studies have shown that it has detrimental consequences.

Due to the weakness and irregularity of the monitoring of AMD impacted locations, the majority of AMD originating in lower middle income to low income countries posed substantial environmental and health risks to the surrounding community. There were various locations impacted by acid mine drainage in the Philippines alone, and these incidents typically occurred in far-flung locales and remote provinces. As a result, it lacked the necessary technology and resources to monitor the amount of heavy metal (HM) content in its water resources on a regular basis. These could pose several concerns, as it was determined that good education and awareness with respect to HM in water resources are vital. A lack of understanding about this condition will result in harmful repercussions not only with respect to the environment but also to the community’s inhabitants. Moreover, it was also discovered that inadequacies in environmental quality monitoring are attributable to a lack of knowledge about the dangers of HM to humans [13]. Chronic exposure to the various HM included in AMD may have serious health consequences. Chromium has been shown to have adverse effects on the liver, kidneys, circulatory system, and neurological system [14]. Exposure to cadmium has been linked to renal impairment, lung illness and cancer, bone abnormalities, kidney damage, and gastrointestinal disorders [15]. Chronic exposure to elevated concentration of iron (Fe) may result in iron toxicosis which is more prevalent in children owing to their increased exposure to iron-containing products. Iron toxicosis may result in gastrointestinal bleeding, diarrhea, hypertension, lethargy, tachycardia, necrosis of the liver, and metabolic acidosis [16]. Manganese poisoning or contact with the body may harm the neurological system and central nervous system [17]. Nickel exposure has been linked to allergic contact dermatitis, respiratory cancer, and reproductive damage [18]. Lead exposure may result in damage to the liver, kidneys, and gastrointestinal tract, as well as acute or chronic neuro-logical impairment [19]. Zinc poisoning may cause harm to the neurological system, while copper toxicity can result in liver and kidney damage, as well as stomach and intestinal discomfort [20,21]. Due to these detrimental effects of HM in AMD, proper and frequent monitoring in affected sites should be implemented. This should be performed in order to ensure that enough information and warnings are provided to the surrounding community.

The most frequent form of monitoring tool is concentration maps. Spatial interpolation is the process of predicting the results of a main component at points within the same area of sample sites [22]. These maps were made with spatial data containing variety of characteristics needed for mapping and characterization to predict water quality in each location. However, spatial data are rarely available, and information on water quality is mainly obtained by spot sampling. Moreover, these techniques are tedious and require expensive instruments, tools, and/or devices. There is no clear evidence on how the performance of spatial prediction methods is affected in existing research, making it impossible to choose the best method for any dataset [23]. Several research studies investigated the performance of several geo-statistical and spatial interpolation techniques in soil moisture and drought [24], PM2.5 estimations [25], wind data [26], digital elevation model (DEM) height accuracy [27], soil organic carbon [28], and even social science [29].

The use of cutting-edge tools such as machine learning (ML) approaches coincided with the transformation of various disciplines to Industry 4.0. ML has opened up new possibilities for unraveling, measuring, and comprehending data-intensive processes in the environment. ML is a unique combination of Big Data technologies and high-performance computers. ML’s overall purpose is to discover patterns in data that inform how problems that are not visible are addressed [30,31]. Numerous studies integrated a ML technique to spatial interpolation models such as Artificial Neural Network (ANN) for solar radiation estimation [32]; deep learning for seismic intensity [33]; ensemble ANN for atmospheric studies [34]; decision tree (DT) approach for land cover data and sodium absorption [35,36]; support vector machine (SVM) for basin precipitation [37]; long short-term memory (LSTM) neural network for PM2.5 [38]; extremely randomized trees for meteorological drought forecasting [39]; support vector regression (SVR) and correlation-based feature selection (CFS) for vehicular emissions prediction [40]; stochastic gradient boosting, cubist, random forest (RF), and model averaged neural networks for temperature maps [41]; random forest for solar radiation observation [42]; ensemble prediction approach for lake acidity prediction [43]; RF and generalized boosted regression (GBR) for soil organic carbon [44]; Non-linear AutoRegressive eXogenous (NARX) model for groundwater (GW) level prediction [45,46,47]; dynamic and long-term prediction of toxic HM [48,49]; and water quality prediction [50]. The use of ML integrated with spatial interpolation technique qualifies as an innovative superior substitute for traditional data application approaches due to its capability to distinguish non-linear associations among numerous constraints as opposed to other techniques that assume these connections are linear [51].

The spatial interpolation methods and its application to environmental monitoring were developed and established already, while the use of ML techniques is currently emerging and growing strongly. Several studies used ML algorithms and have recently been integrated to spatial interpolation. Du et al. investigated the suitability of various ML techniques for spatial data management. It addressed associated issues such as non-linear and high-dimensional classification and regression utilizing semi-supervised and active learning. This is to manage limited training data sizes and identify high-level features in the dataset. The findings of the research of Du et al. indicated that ML techniques are appropriate for overcoming these difficulties in spatial data processing that enhance the performance of classification and prediction skills of the model [52]. Moreover, the SVR and RF were the most widely explored ML technique in reference to its integration to spatial interpolation [53].

According to Stahl et al., the sample density influences the performance of the spatial interpolation technique. When the sample density is low, the performance of spatial interpolation technique is deficient. However, when sample density is high, the performance of spatial interpolation technique improves [54]. Spatial data are rarely available, and the majority of information on GW quality is gathered via spot sampling. Hence, the spatial interpolation technique is useful in GW quality monitoring and mapping. Therefore, this research addresses the issue in sampling density and the challenge of spot sampling access by highlighting the application of a hybrid neural network with particle swarm optimization (NN-PSO) with the spatial interpolation technique for GW quality mapping.

## 2. Materials and Methods

The study uses NN-PSO methodology relative to a range of interpolation types, including deterministic techniques, geostatistical methods, and interpolation with barriers, to improve its GW quality prediction performance. The subsequent sections detail the study’s focus and the combined strategies used to create enhanced spatial maps.

### 2.1. The Area of Study

The Province of Marinduque, the smallest island province in the MIMAROPA Region (or Region IV-B), is the area of research. Marinduque Island, about 200 kilometers south of Manila, is a province known as Philippines’ heart due to its geometric shape and geographical location [55]. With a total land area of 96,000 hectares, Marinduque is a 4th income class island province made up of six municipalities: Boac, Buenavista, Mogpog, Gasan, Santa Cruz, and Torrijos. The province’s topography is mostly mountainous, with continuous and severe slope areas. Considering the province’s overall land area, 77 percent, or 737.2 square kilometers, is classified as alienable land, while the remaining 23 percent, or 222.05 square kilometers, is classified as forest land [56]. The island province has a Climate Type III climate, with the dry season lasting from November to April and the wet season covering the rest of the year [57]. The annual rainfall in the province of Marinduque range from 1700 to 2500 mm [58].

Several bodies of water, primarily rivers and their tributaries, make up the province of Marinduque. The province’s major rivers and tributaries have a total length of 178 kilometers which is composed of the municipalities of Boac (20.11 percent), Buenavista (10.96 percent), Gasan (19.49 percent), Mogpog (20.06 percent), Santa Cruz (15.22 percent), and Torrijos (15.22 percent) (14.16 percent). The province has a total of 614.1003 km^2^ considering the drainage area. The Municipality of Boac has 34.80 percent of the total surface water body drainage area, followed by 7.47 percent in the Municipality of Buenavista, 11.30 percent in the Municipality of Gasan, 14.61 percent in the Municipality of Mogpog, 19.58 percent in the Municipality of Santa Cruz, and 12.24 percent in Torrijos [59].

**Figure 1:**
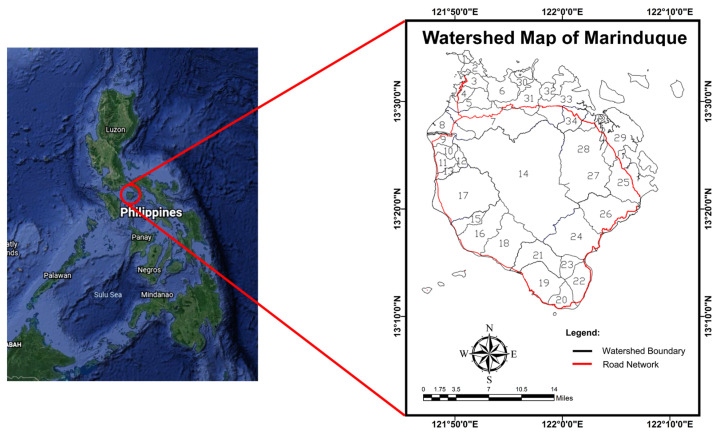
Watershed Map of Marinduque.

Figure 1 depicts 34 watersheds that make up the province of Marinduque. With an extent of 195.94 km^2^, the Boac Watershed is the province’s largest watershed. The list of watersheds of the province of Marinduque is shown in Table 1.

Marinduque Island is home to one of the Philippines’ largest copper deposits. Since 1969, copper mining activities have been carried out on the island. Mine tailings from these activities began to be deposited in Calancan Bay in 1975. Since then and until 1997, around 200 million tons have been dumped [60].

In the 1990s, the island was hit by two mining catastrophes. The first incident occurred in 1993, when the Maguilaguila siltation dam in San Antonio, Sta. Cruz, collapsed. This caused property and agricultural damage and adverse effects to public health in downstream communities [61]. Three years later, in 1996, the Tapian Pit collapsed, releasing between 180,000 and 260,000 cubic meters of mine tailings into the Boac River, causing environmental and community damages [62].

### 2.2. Sampling, Storage, and Collection of GW Samples

**Figure 2:**
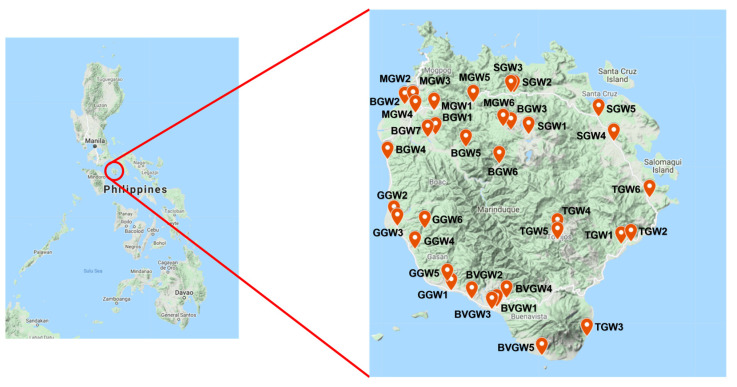
Map of the Sampling Locations.

GW samples were gathered from different wells in six municipalities in Marinduque and stored in plastic bottles (1 L). Data were collected and stored in compliance with EPA No. SESDPROC-301-R3, which is the GW sampling operational procedure [63]. The Hanna HI 9811-5 handheld multi-parameter sampler was used to collect all field measurements, including in situ physicochemical parameters for GW samples, such as temperature (in Celsius), pH, total dissolved solids (TDS) in milligrams per liter, and electrical conductivity (EC) in microsiemens per centimeter. Two separate geographical maps were created to depict dry and wet seasons in a year [64]. The map and details of the sampling locations are presented in Figure 2 and Table 2.

### 2.3. Elemental Analysis of Groundwater Samples

The measurement of total metals, which includes suspended and dissolved components as well as soluble metals, is required when looking for HMs in GW samples. The EPA Method 3005A was employed for Inductively Coupled Plasma spectroscopy using water acid digestion for total dissolved and recoverable metals as the reference guidelines for GW sample digestion [65,66].

### 2.4. Descriptive and Multivariate Statistical Analysis

The IBM Statistical Package for the Social Sciences (SPSS) was utilized to evaluate descriptive statistics linked to GW physicochemical parameters and HM intensities. Skewness and kurtosis were applied to assess the asymmetry of physicochemical characteristics and HM concentrations in GW. The skewness of the GW quality parameters shows the relative locations of the median and mean, whereas the kurtosis represents the form of the distribution [67,68]. The most important element in characterizing the variability of GW physicochemical parameters and HM content was the coefficient of variation (CV). The coefficient of variability was used to analyze the dataset’s variability as follows: CV ≤ 15%, low; 15% < CV ≤ 35%, intermediate; and CV ≥ 35%, high [69]. Moreover, a Kolmogorov–Smirnov (K-S) test was employed to test the normality of the datasets and to examine if the GW quality parameters have a normal distribution [70].

The GW quality data are frequently identified and evaluated using multivariate statistical analysis. Multivariate statistical approaches enable the extraction of meaningful meaning from data by simplifying, organizing, and classifying it [71]. Using a correlation matrix utilizing MATLAB 2021a and R studio, the relationship between physicochemical parameters and HM intensities in GW in the research area was observed. The correlation matrix established occurrence, HM associations, and potential source of contaminants in the area of study [72]. The R value of −1 signifies that the parameter shifts inversely with respect to the other. A very strong correlation is exhibited by 0.90 < r < 1.00, strong correlation by 0.70 < r < 0.89, moderate correlation by 0.40 < r < 0.69, weak correlation by 0.10 < r < 0.39, and negligible correlation by 0 < r < 0.10. There is no association between the two variables if the correlation is zero [73].

### 2.5. Machine Learning: Hybrid Neuro-Particle Swarm Optimization Modelling

Machine learning is an application of artificial intelligence that allows software applications and uses statistical models and algorithms to analyze and draw interferences from patterns in data. This study uses MATLAB 2021a to enhance the prediction capability of the spatial interpolation maps of GW quality using a Particle Swarm Optimization (PSO) trained Artificial Neural Network model. Subsequent sections below elaborate how machine learning was used as technique in creating GW quality mapping. Hence, pages 7–9 discuss and illustrate how machine learning has been used in the study and which stage of the technique development been used.

#### 2.5.1. Backpropagation Neural Network (BP-NN)

**Figure 3:**
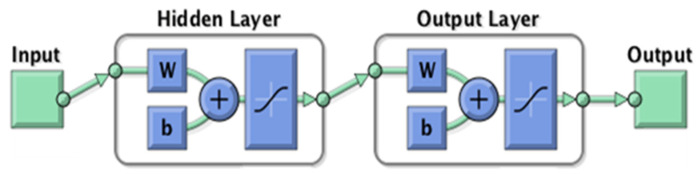
Architecture of the ANN showing the weights and biases.

The ANN is an approach inspired by a real biological neuron that has been used in prediction and forecasting, particularly for complicated and non-linear systems such as environmental problems such as water quality modeling [74]. The Artificial Neural Network learns by training the connectivity between the neurons, which is performed using known input and output values provided in an organized manner so that the network can extract the relationship and patterns in the dataset [75]. MATLAB R2021a was used to create the neural network model, which included 70 percent, 15 percent, and 15 percent data partitioning for the data sets utilized in the training, validation, and testing stage, respectively [76]. The Levenberg–Marquardt algorithm was employed as the model’s training algorithm since it is the quickest method to train a moderate-sized feed forward neural network with several hundred weights [77]. The model uses a hyperbolic tangent sigmoid (tansig) transfer function as the driving component for the interaction between a neuron’s weights (W) and the input element. It also has a significant impact on the network’s complexity and performance, and it was chosen because it provides ideal decision biases (b). The tansig transfer function can understand the complex non-linear connection between the input and output parameters by producing values ranging from −1 to +1 [78]. Figure 3 depicts the design and architecture of the ANN model development.

#### 2.5.2. Particle Swarm Optimization (PSO)

One of the most frequently used ANN models is the BP-NN, which is a multiple layer feed-forward ANN trained using the error BP method. BP-NN, on the other hand, has weaknesses, which may be fixed by combining it with the Particle Swarm Optimization (PSO) approach, which improves the model’s accuracy and efficiency [79]. The PSO approach is utilized in this research in order to optimize the link weights of the ANN.

The PSO is a type of swarm intelligence approach used in evolutionary computing. These techniques were influenced by natural bio-social phenomena such as flocks of birds, schools of fish, and other natural bio-social phenomena. The PSO is particularly applicable for non-linear generalization capabilities with discontinuities because of its quick convergence and robustness. PSO is a promising choice for optimization modelling when compared to other evolutionary algorithms [80,81]. Due to its faster learning speed and lower memory demand, the PSO is preferred over other optimization algorithms including the Genetic Algorithm (GA) and Imperialist Competition Algorithm (ICA) [82].

#### 2.5.3. Hybrid NN-PSO Model

**Figure 4:**
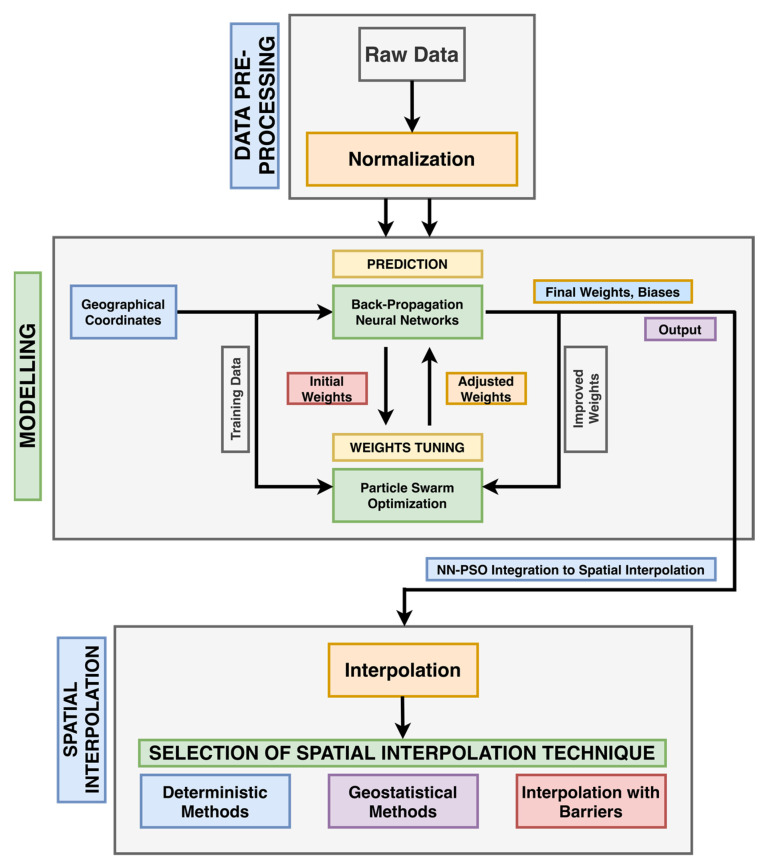
Schematic diagram of the NN-PSO informed spatial interpolation.

The Neural Network (NN) model’s connection weights are optimized via Particle Swarm Optimization (PSO). The PSO is utilized because it can determine the best solution while also reducing the ANN’s errors. The PSO calculates the positions of the particles and transmits it to the learning process. The optimal weights and biases for the training method of the ANN were found using the PSO-generated particle population [83]. The framework of the application of the NN-PSO method in spatial interpolation techniques is presented in Figure 4.

#### 2.5.4. Performance Evaluation

The correlation coefficient (R) and mean squared error (MSE) were utilized to assess the performance of the NN-PSO model. A complete positive correlation is implied by a R value of 1. The R value indicates how closely two variables were linked [84]. The R values were observed and utilized as performance indicators throughout the validation and testing phases. The R value was utilized in the validation phase to assess network generalization, terminate the simulation when generalization ceased to improve, and determine the optimum architecture, while the R value in the testing phase serves as an additional independent measure of network performance during and after the simulation [85,86]. In a simulation using the NN-PSO method, the MSE was minimized which includes the overall MSE in the validation and testing phases. The MSE is a helpful tool for assessing model predictions since it reflects the sum of squared bias and variance. For the model, zero is the optimum value for the MSE [87]. The equations for the R and MSE are shown in Equations (1) and (2) where “*N*” is the number of data sets, *y*_0_ is the predicted value, *y_m_* is the measured value, $\bar{\bar{y}}$ and $\overset{‗}{\overset{‗}{y_{m}}}$ were the mean values, and *e_i_* is the contrast between the measured values and the predicted values [88].

(1) $$r=\frac{\sum_{i=1}^{N}\left(y_{0}-\bar{y}\right)\left(y_{m}-\bar{y_{m}}\right)}{\sqrt{\sum_{i=1}^{N}{\left(y_{0}-\bar{y}\right)}^{2}\sum_{i-1}^{N}{\left(y_{m}-\bar{y_{m}}\right)}^{2}}}$$

(2) $$MSE=\frac{1}{n}\sum_{i=1}^{N}{\left(e_{i}\right)}^{2}$$

### 2.6. Spatial Interpolation Methods for Heavy Metals

The measured physicochemical parameter and detected HM concentrations were mapped by utilizing the ArcGIS platform. The sampling sites’ precise locations were recorded using a GARMIN Montana 650 GPS, which was integrated onto the Geographical Information System (GIS) platform. Moreover, data collected from the GW samples were applied to create maps operating the Geostatistical Analyst Tool and Geostatistical Wizard in the ArcGIS software. The different interpolation methods are utilized to apply spatial analysis which are included in the ArcGIS spatial analysis extension tool. Deterministic techniques, geostatistical methods, and interpolation with barriers are among the three (3) interpolation types utilized in the study.

The deterministic techniques include Inverse Distance Weighting (IDW), Global Polynomial Interpolation (GPI), Radial Basis Functions (RBF), and Local Polynomial Interpolation (LPI). The IDW is a deterministic spatial interpolation approach that determines the data in an unsampled location by using the data from a distributed collection of sampled locations. The data in an unidentified site are based on the weighted sum of the values of the recognized locations which is based on the distance of the unidentified site to the sampled locations [89]. GPI is a deterministic and approximate trend surface analysis wherein a smooth two-dimensional polynomial function of first, second, or higher degree is used to describe a surface. It computes the target point’s value by using all nearby points [90]. RBF achieves its accuracy by using a large number of accurate interpolators that reduce the overall curvature of the surface depending on the space between the interpolated and sampled locations [91]. The LPI method includes fitting the weighted least squares to store the data inside the search ellipse of the grid node wherein the projected value is used to calculate the surface value of the neighboring points that can be used to build surface that account for short-range variation [92].

The geostatistical techniques include Ordinary Kriging (OK), Universal Kriging (UK), and Empirical Bayesian Kriging (EBK). OK is a linear geostatistical process that depends less on stationary mean assumptions by using the search radius. The OK method approximates values in unsampled regions by averaging nearby data and visualizing the correlations between surrounding values as a function of the geographic distance between the sites in the area of study using a weighted average of neighboring data and a variogram [93]. UK uses a trend surface that may include factors that account for variation in the global component, and it more likely to provide residuals that are more closely related to a stationary mean with identical distribution [94]. EBK automates the most time-consuming and challenging stages in creating a realistic kriging model. EBK automatically optimizes by subsetting and simulating several semi-variogram models instead of a single semi-variogram. EBK creates a semi-variogram model from existing data and then simulates the new value at each input data point until the final calculation of the new semi-variogram model based on the simulated data [95].

The interpolation with barriers includes Kernel Smoothing (KS) and Diffusion Kernel (DK). The DK employs a complex distance metric specified by the cost surface, which is a widely used raster function that estimates the cost of traveling from one cell of a raster to the next, and then generates forecasts on automatically chosen grids. The KS method is a variation of first order LPI that avoids computing uncertainty by using a technique similar to that used to estimate regression coefficients in ridge regressions. KS utilizes the shortest distance between locations to connect places on opposing sides of an absolute barrier using a sequence of straight lines [96]. These interpolation techniques were implemented to the data arrays in order to distinguish the concentration map that best describes the HM pollution in the province of Marinduque [97].

### 2.7. Cross Validation

A frequently utilized technique for comparing the interpolation methods is cross validation. Due to the small sample size, cross validation was used. A cross validation procedure consists in removing data points one at a time, interpolating a value from the remaining observations, and comparing that value to the real value [98].

The Mean Absolute Error (MAE) and Pearson’s Correlation Coefficient (R) were used to determine the predictive accuracy of distinct methods, with the least MAE representing the most exact predictions. Equation (3) shown below is used to calculate MAE:

(3) $$MAE=\frac{1}{n}\sum_{i=1}^{n}\left|Z_{i}-Z\right|$$

where *Z_i_* is the predicted value, *Z* is the observed value, and *n* is the number of observations [99].

## 3. Results

The major findings of the study are presented in this section. This section contains all the maps created with the integrated NN-PSO and spatial interpolation algorithms. This part also includes the data’s descriptive statistics as well as the maps’ prediction performance.

### 3.1. Heavy Metals in Groundwater

Table 3 shows descriptive data for GW physicochemical characteristics and HM concentrations.

During the dry season, all GW physicochemical parameters and HM concentrations were observed to be highly variable, except for pH and temperature, which were found to be low and moderately variable, respectively. The Kolmogorov–Smirnov test revealed that the dry season physicochemical characteristics and HM concentrations of GW in the research region were not uniformly distributed, with all parameters having *p* < 0.05.

The GW physicochemical characteristics were compared to Philippine National Standards for Drinking Water (PNSDW) 2017 and World Health Organization (WHO) Drinking Water Standards. Table 3 shows that the pH of the GW during the dry season ranged from 6.10 to 7.90, with an average pH of 7.02, which is within the PNSDW 2017 and WHO standards. The EC of the GW during the dry season varies from 80 to 2350 µS/cm with mean EC of 935.17 µS/cm, which is below the maximum value set by the WHO. The asymmetries of the physicochemical characteristics and HM concentration of the GW during the dry season were measured by skewness and kurtosis. With the exception of pH, all physicochemical characteristics and HM concentrations in GW exhibit a positive skewness, meaning that the right side is longer than the left. This indicates that an asymmetry distribution with a positive skewness tends to be less concentrated. TDS, Cr, Fe, Mn, and Zn are the only elements with positive kurtosis values, indicating a steeper distribution than normal.

A comparative assessment of HM concentrations of GW during the dry season was likewise performed relative to the PNSDW 2017 and WHO Standards for Drinking Water. Mean concentrations of Ni ranging from 0.000110 to 0.125310 ppm and Cu ranging from 0.000868 to 0.260497 ppm were below the limit of PNSDW 2017 and WHO standards for drinking water. Zn concentration ranged between 0.000985 and 56.96133 ppm, with mean concentration exceeding the WHO limit. Average concentrations of Cr, Cd, Fe, and Pb exceeded the allowable limits by PNSDW 2017 and WHO Standards. Moreover, some locations had concentrations way above the limit.

During the wet season, all GW physicochemical parameters except for pH and temperature and HM concentrations showed considerable variability. The Kolmogorov–Smirnov test revealed that the wet season physicochemical characteristics and HM concentrations of GW in the research region were not normally distributed, with *p* < 0.05 for all parameters.

Wet season GW physicochemical characteristics were also compared to PNSDW 2017 and WHO Drinking Water Standards. Table 4 indicates that the pH of the GW throughout the wet season ranged from 6.00 to 9.55, with a mean pH of 7.43, which is within the PNSDW 2017 and WHO standards.

The EC of the GW during the wet season varies from 70 to 2640 µS/cm with average 780.61 µS/cm which is inside the acceptable value set by the WHO. The asymmetries of the physicochemical characteristics and HM concentration in GW during wet season were measured by skewness and kurtosis. With the exception of Cr, Cd, Pb, and Cu, all physicochemical characteristics and HM concentrations in GW exhibited positive skewness, meaning the right side is longer than the left side. This indicates that an asymmetry distribution with a positive skewness tends to be less concentrated. All parameters have a negative kurtosis value, which suggests that the distribution of the datasets was flatter than a normal distribution.

The HM concentrations in GW during the wet season were assessed in comparison to the PNSDW 2017 and WHO Standards for Drinking Water. Only the mean Ni content was below the PNSDW 2017 and WHO drinking water requirements. Cr, Cd, Fe, Mn, Pb, Zn, and Cu values were above the permissible limits set by PNSDW 2017 and WHO. Furthermore, some sites have concentrations that were far higher than the permissible level.

A Pearson’s Correlation Matrix (PCM) was utilized to establish the level of correlation between GW HMs and physicochemical properties in the island province of Marinduque, with the goal of identifying a potential source of the HMs. Table 5 shows the metals correlation matrix that was generated during the dry season. Cd (r = 0.72), Ni (r = 0.69), and Pb (r = 0.81) were all highly linked with chromium. Cadmium and Ni (r = 0.78), Cd and Pb (r = 0.83), and Ni and Pb (r = 0.77) showed substantial positive associations. Positive significant correlations imply that these metals have a shared origin, were mutually dependent, and behaved similarly throughout transport [100].

**Figure 5:**
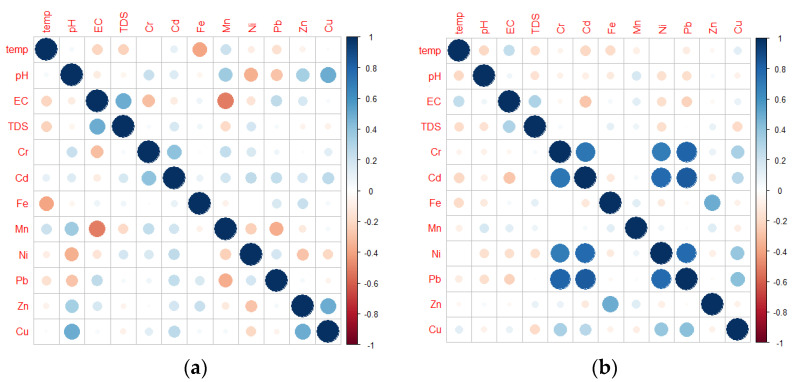
Correlation matrix plot of GW in the study area during (**a**) dry and (**b**) wet season.

The correlation analysis for water quality parameters during the wet season is shown in Table 6 and illustrated in Figure 5. For the physicochemical characteristics, the EC was observed to have a positive correlation to TDS which agreed to the findings of Manikandan et al. in 2020 [101]. A significant connection was found between Cr and Cd, as well as Cu and Zn, for the HMs in GW during the wet season. This indicated a possible shared source for these HMs. Moreover, these correlations were in agreement with the findings of Kumar et al. in 2012 [102] and Mansouri et al. in 2012 [103].

### 3.2. NN-PSO Simulation Results

The hybrid NN-PSO was utilized to improve the efficiency and robustness of spatial interpolation mapping of GW quality. The proposed method was evaluated by considering different internal characteristics of the network. The Levenberg–Marquardt method was used as the training algorithm, and the hyperbolic tangent sigmoid was used as the transfer function for the input layer (IL) to hidden layer (HL) and HL to output layer (OL) in the hybrid NN-PSO informed spatial interpolation approaches. The results of the NN-PSO simulation for the dry and wet season GW quality parameters are presented in Table 7 and Table 8.

**Figure A1:**
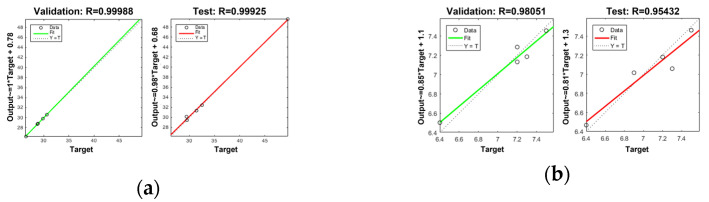
R value plots for validation and testing phase of the NN-PSO: (**a**) dry season temperature; (**b**) dry season pH.

**Figure A2:**
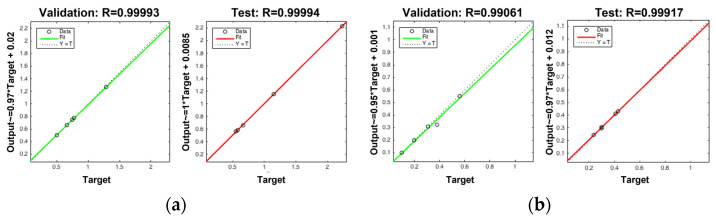
R value plots for validation and testing phase of the NN-PSO: (**a**) dry season EC; (**b**) dry season TDS.

**Figure A3:**
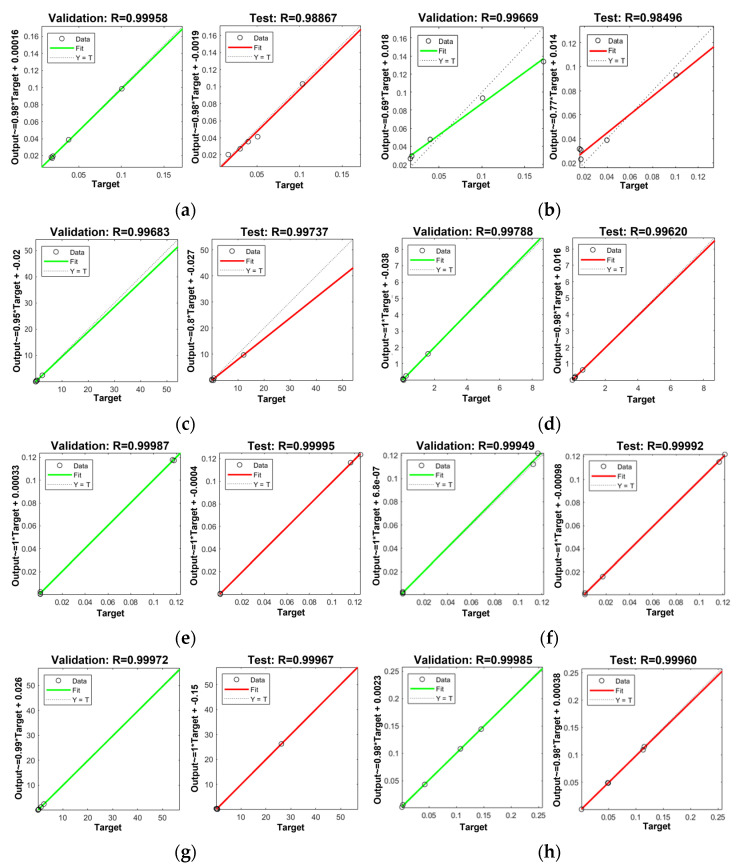
R value plots for validation and testing phase of the NN-PSO: (**a**) dry season Cr; (**b**) dry season Cd; (**c**) dry season Fe; (**d**) dry season Mn; (**e**) dry season Ni; (**f**) dry season Pb; (**g**) dry season Zn; (**h**) dry season Cu.

**Figure A4:**
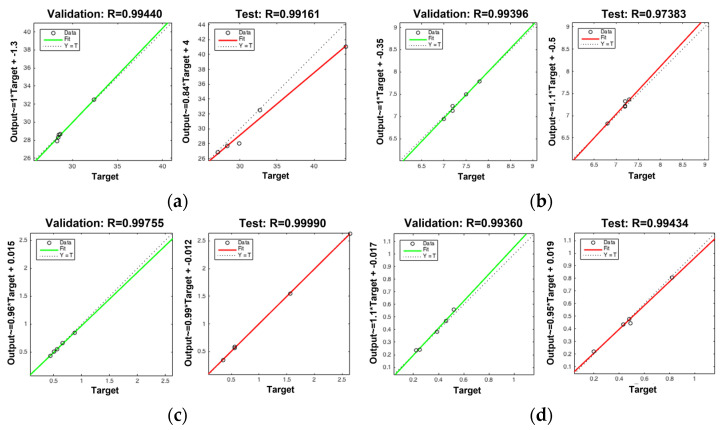
R value plots for validation and testing phase of the NN-PSO: (**a**) wet season temperature; (**b**) wet season pH; (**c**) wet season EC; (**d**) wet season TDS.

**Figure A5:**
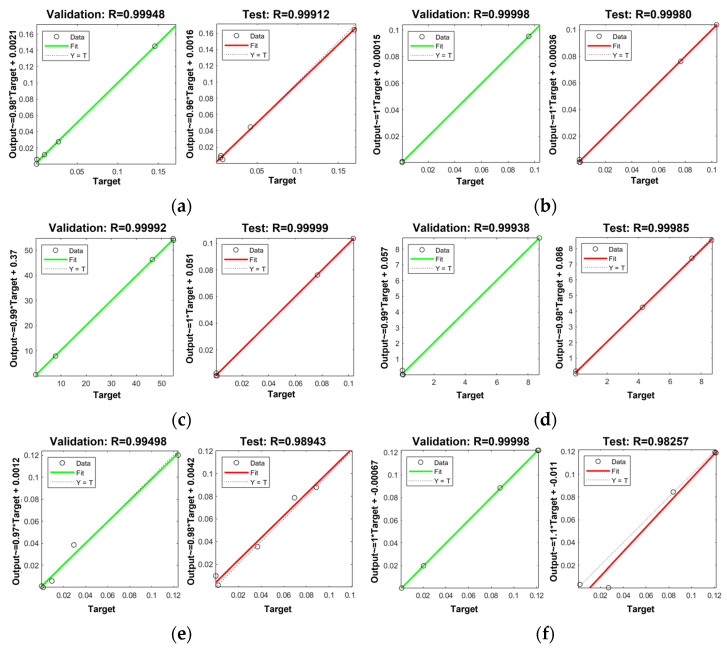
R value plots for validation and testing phase of the NN-PSO: (**a**) wet season Cr; (**b**) wet season Cd; (**c**) wet season Fe; (**d**) wet season Mn; (**e**) wet season Ni; (**f**) wet season Pb.

**Figure A6:**
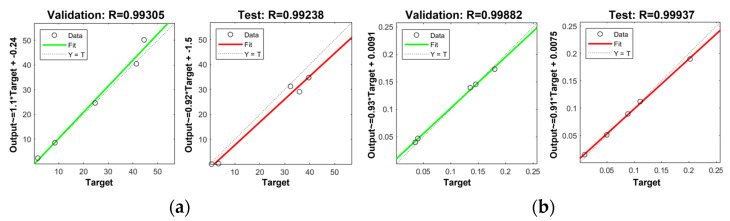
R value plots for validation and testing phase of the NN-PSO: (**a**) wet season Zn; (**b**) wet season Cu.

The results and efficiency of the NN-PSO simulation for dry and wet season GW quality parameters showed excellent results, as evidenced by the extremely low MSE (ideal value is zero) and extremely high R values for internal validation and testing of the NN-PSO models (ideal value is one). These NN-PSO models informed the spatial interpolation techniques which improved the accuracy and performance of the GW quality maps. The R plots of the NN-PSO simulation for the physicochemical parameters and HM concentrations during the dry and wet season are presented in Figure A1, Figure A2, Figure A3, Figure A4, Figure A5 and Figure A6 in Appendix A.

### 3.3. NN-PSO Informed Spatial Interpolation Techniques for GW Quality Mapping

**Figure 6:**
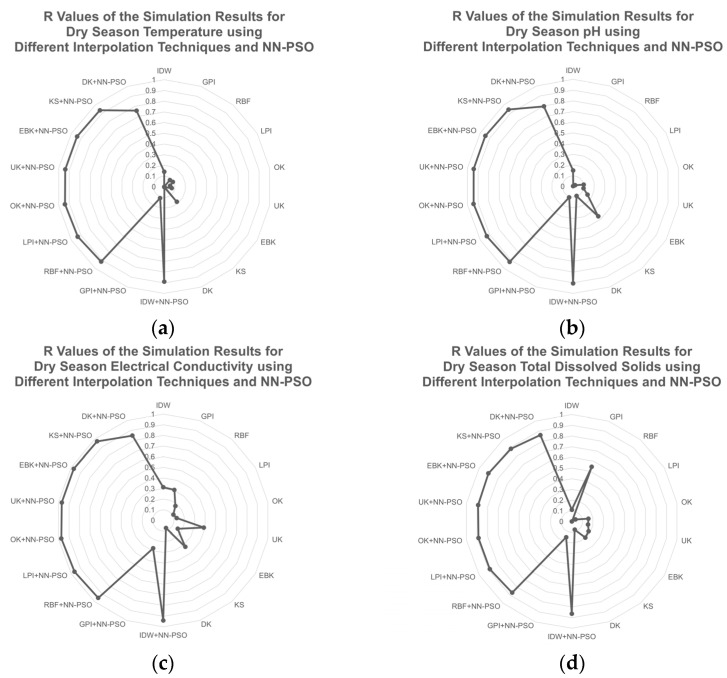
R values of the simulation results for the physicochemical characteristics during dry season using different interpolation techniques and NN-PSO: (**a**) temperature; (**b**) pH; (**c**) EC; and (**d**) TDS. These figures illustrate the performance and efficiency of interpolation techniques for mapping the physical properties of GW during the dry season.

**Figure 7:**
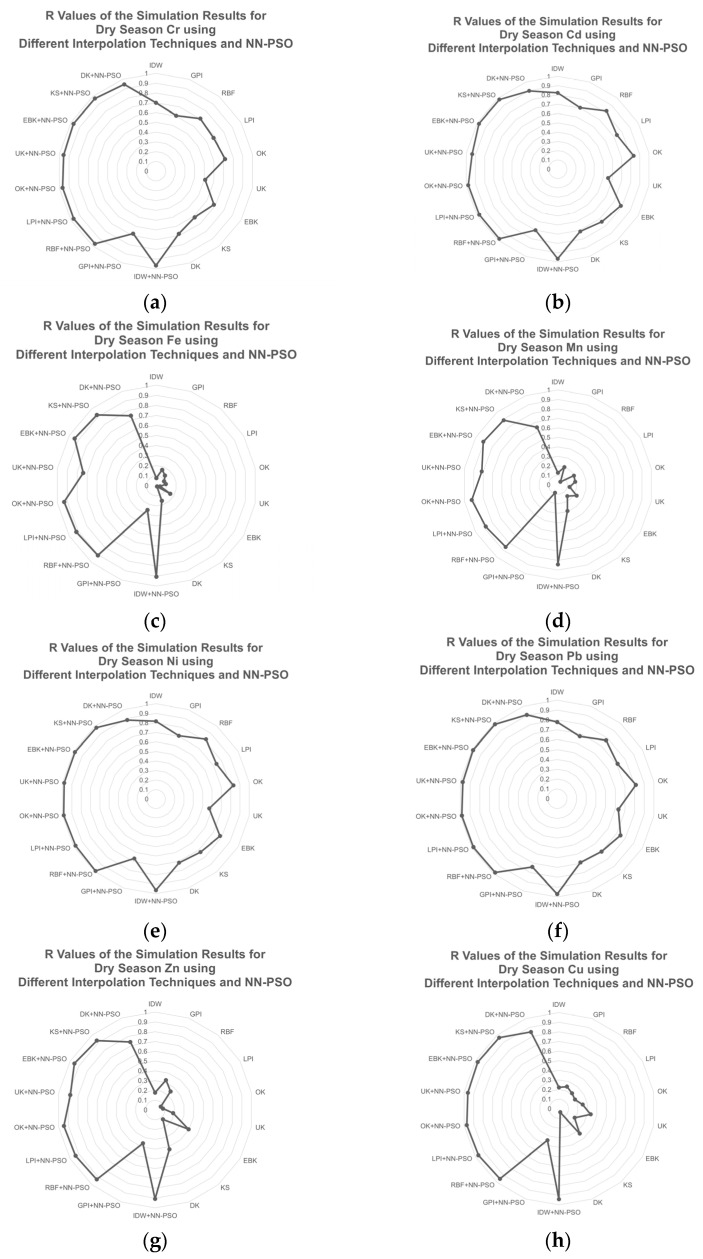
R values of the simulation results for HM concentrations during dry season using different interpolation techniques and NN-PSO: (**a**) Cr; (**b**) Cd; (**c**) Fe; (**d**) Mn; (**e**) Ni; (**f**) Pb; (**g**) Zn; and (**h**) Cu.

The performance of the NN-PSO informed spatial interpolation approaches for GW quality mapping was evaluated via cross validation, with MAE and R values utilized in order to assess the prediction capability of the various interpolation techniques. The approach that produced the lowest MAE value and the greatest R value was regarded the best. The accuracy and effectiveness of the techniques employed to interpolate GW quality during the dry season are displayed in Figure 6 and Figure 7. The complete values for the performance of the different interpolation techniques during the dry season are shown in Table A1 of Appendix B.

Various interpolation techniques were applied in order to evaluate the spatial variability of GW quality in the province of Marinduque during the dry season, including IDW, GPI, RBF, LPI, OK, UK, EBK, DK, and KS. Moreover, these interpolation techniques were likewise assessed after being informed and integrated with NN-PSO.

Physicochemical properties and HM concentrations of GW were mapped and analyzed using various interpolation approaches throughout the dry season. Except for Nickel (Ni), which had the best mapping prediction performance using NN-PSO informed radial basis functions, NN-PSO informed geostatistical techniques and OK and EBK methods, in particular, were the best approaches for mapping the GW quality during the dry season. The performance of the best interpolation technique was manifested through its lowest MAE and highest R values.

Figure 6 illustrates that the NN-PSO informed OK model had the best mapping prediction for GW temperature and EC during dry season. The OK-NN-PSO method had the least MAE and highest R value which is significantly higher than the best method observed without NN-PSO. The EBK-NN-PSO method was observed to have the best prediction performance for groundwater pH and TDS as evident to its lowest MAE and highest R value. Similarly, these validation criteria have significant improvement than compared to the interpolation methods that were not NN-PSO informed.

During the dry season, HM concentrations in GW were also mapped and evaluated by using several spatial interpolations approaches as shown in Figure 7. Using the NN-PSO informed Empirical Bayesian Kriging technique (EBK-NN-PSO), the best mapping prediction performance was found for Cr, Cd, Fe, Pb, Zn, and Cu.

**Figure A7:**
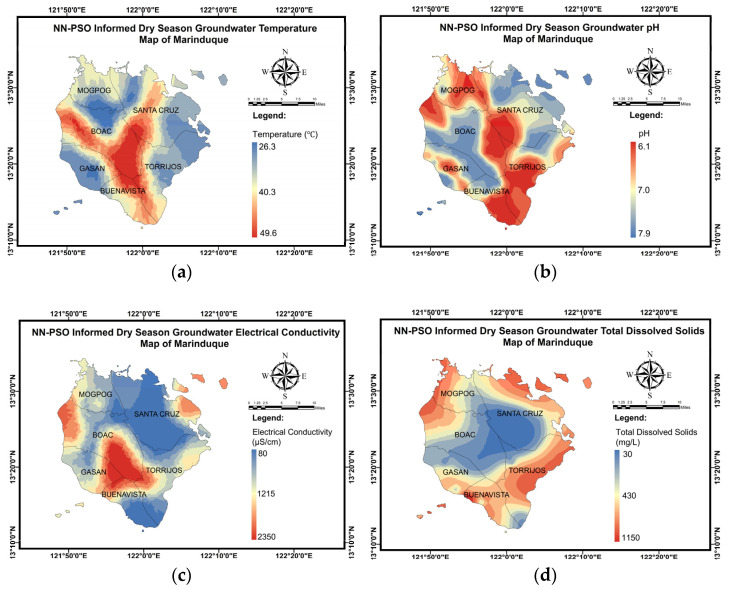
NN-PSO informed maps of the GW physicochemical characteristics during dry season: (**a**) temperature; (**b**) pH; (**c**) EC; (**d**) TDS.

**Figure A8:**
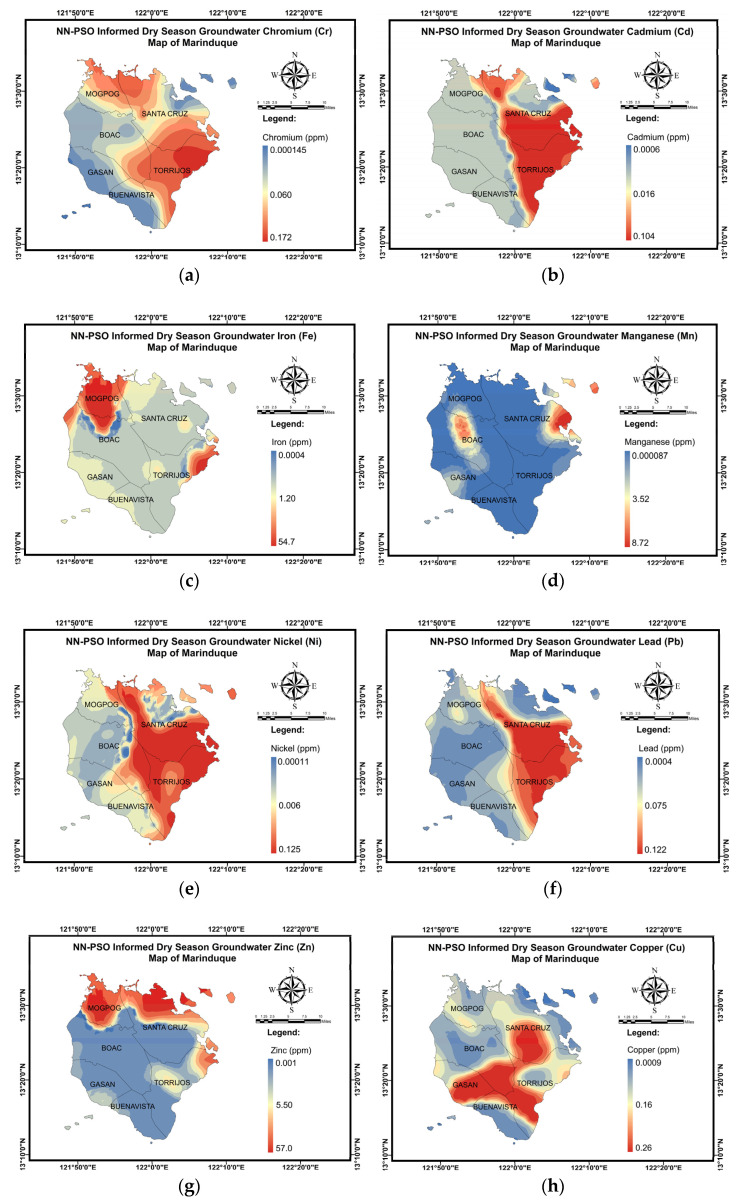
NN-PSO informed maps of the GW heavy metal concentration during dry season: (**a**) Cr; (**b**) Cd; (**c**) Fe; (**d**) Mn; (**e**) Ni; (**f**) Pb; (**g**) Zn; (**h**) Cu.

Among the interpolations employed, the EBK+NN-PSO technique for mapping Cr, Cd, Fe, Pb, Zn, and Cu has the lowest MAE. The R value of the NN-PSO informed EBK method used for Cr increased by 35.61%, Cd = 17.91%, and Pb = 21.65%. Furthermore, R values of Fe, Zn, and Cu improved by 5.6, 2.4, and 2.9 times, respectively. Manganese (Mn) and Nickel (Ni) were observed to have the best prediction performance using NN-PSO informed OK and RBF method, respectively. Correspondingly, the performance of these models for Mn and Ni was observed to have the lowest MAE among the interpolation techniques utilized and 3.1 and 1.2 times higher than compared to interpolation techniques without NN-PSO. The NN-PSO informed dry season GW physicochemical characteristics and HM concentration map of Marinduque are presented in Figure A7 and Figure A8 of Appendix C.

**Figure 8:**
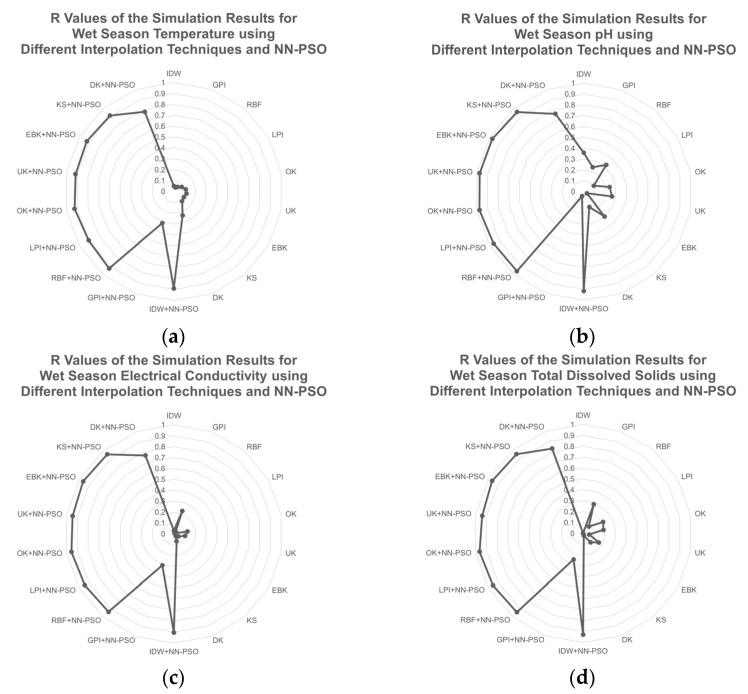
R values of the simulation results for the physicochemical characteristics during wet season using different interpolation techniques and NN-PSO: (**a**) temperature; (**b**) pH; (**c**) EC; and (**d**) TDS.

**Figure 9:**
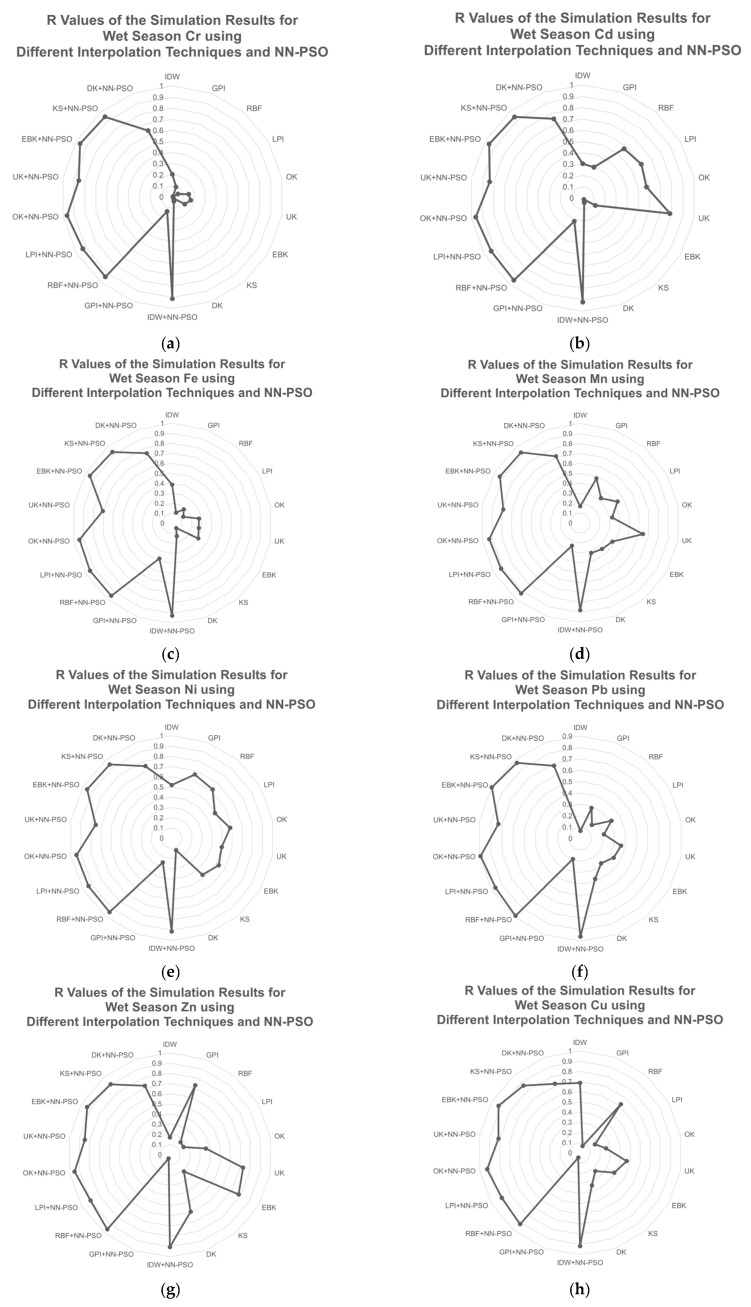
R values of the simulation results for HM concentrations during wet season using different interpolation techniques and NN-PSO: (**a**) Cr; (**b**) Cd; (**c**) Fe; (**d**) Mn; (**e**) Ni; (**f**) Pb; (**g**) Zn; and (**h**) Cu.

The NN-PSO informed geostatistical approaches, comprising OK and EBK, were shown to be the optimum method for the wet season GW physicochemical properties, with the lowest MAE and highest R. For groundwater temperature and pH, OK was the best technique, whereas EBK was the best method for EC and TDS. The NN-PSO informed OK was the best among the interpolation techniques integrated with NN-PSO and had an R observed to be 3.9 times greater than compared to the best interpolation method without NN-PSO. Additionally, the other physicochemical parameters including GW pH, EC, and TDS were observed to have an R value of 2.7, 4.3, and 3.4 times higher than the spatial interpolation methods with NN-PSO integration. The performance of the different spatial interpolation techniques for the parameters observed during the wet season is presented in Figure 8 and Figure 9. As illustrated in Figure 8, the OK+NN-PSO model provided the most accurate mapping forecast for GW temperature and pH during wet season. Among the observed spatial interpolation techniques for temperature and pH, the OK+NN-PSO approach achieved the highest R value and the lowest MAE. The EBK+NN-PSO approach had shown the greatest prediction performance for GW EC and TDS during wet season.

As illustrated by Figure 9, EBK+NN-PSO provided the best mapping prediction performance for Fe, Mn, Ni, Pb, and Zn. OK+NN-PSO provided the best mapping prediction performance for Cr, Cd, and Cu. RBF+NN-PSO provided the best mapping prediction performance for Zn. Table A2 in Appendix B elaborates the complete values for the performance of the various interpolation methods for the wet season.

**Figure A9:**
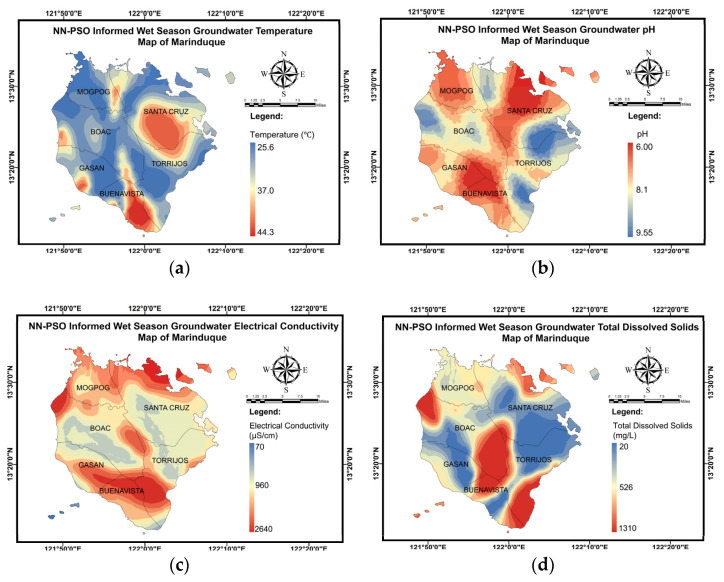
NN-PSO informed maps of the GW physicochemical characteristics during wet season: (**a**) temperature; (**b**) pH; (**c**) EC; (**d**) TDS.

**Figure A10:**
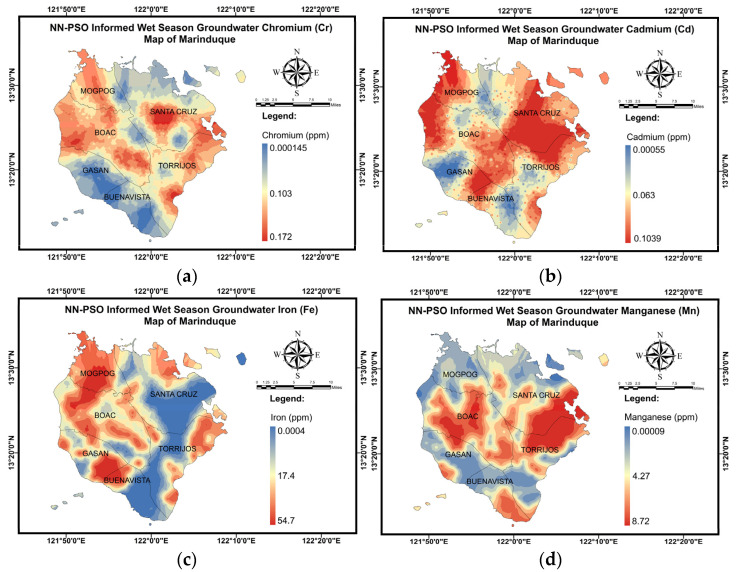
NN-PSO informed maps of the GW HM concentration during wet season: (**a**) Cr; (**b**) Cd; (**c**) Fe; (**d**) Mn.

**Figure A11:**
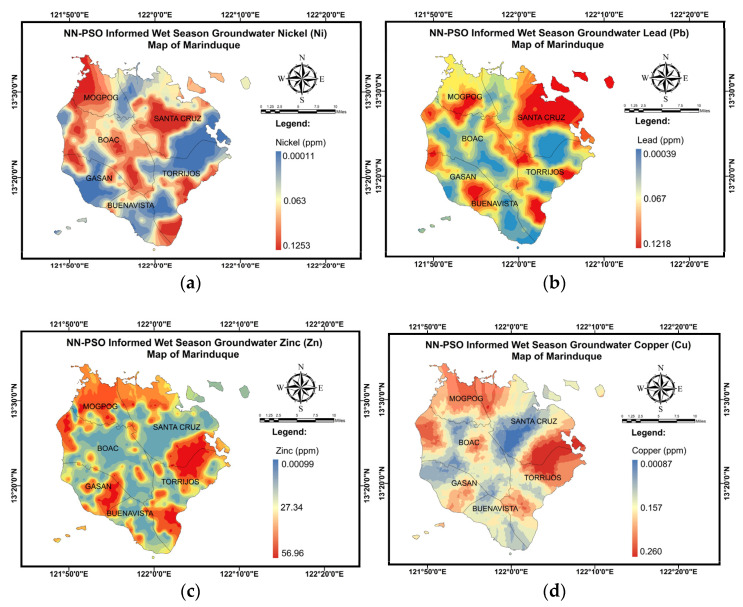
NN-PSO informed maps of the GW HM concentration during wet season: (**a**) Ni; (**b**) Pb; (**c**) Zn; (**d**) Cu.

The HM concentration during the wet season was mapped using the different interpolation techniques with and without the integration of NN-PSO. Chromium (Cr), Cadmium (Cr), and Copper (Cu) concentrations were mapped using the best method observed which is the integration of Ordinary Kriging (OK) and NN-PSO. The OK+NN-PSO technique had the lowest MAE, and its R value was 4.7, 1.2, and 1.3 times greater than the maximum R value found for the interpolation method without NN-PSO integration. The metals Fe, Mn, Ni, and Pb have the best prediction performance based on MAE and R utilizing Empirical Bayesian kriging (EBK) combined with NN-PSO. When compared to interpolation approaches without NN-PSO integration, the EBK-NN-PSO method provided the lowest MAE and R values, which were 2.5, 1.5, 1.4, and 2.4 times higher. The spatial interpolation maps with the best prediction performance for the physicochemical characteristics and HM concentrations are presented in Figure A9, Figure A10 and Figure A11 of Appendix C.

The summary of the cross-validation performance of the models for the physicochemical parameters and the HM concentrations both for dry and wet season is exhibited in Table 9.

## 4. Discussion

Based on the results, the use of the hybrid neural network–particle swarm optimization method in spatial interpolation was able to address the sampling density issue experienced in water quality monitoring. It provides solutions on data gaps when the spatial distribution map of GW quality becomes available by having simple water parameter such as pH [104]. The impact of this research is emphasizes on prediction improvement for mapping specific features and water quality parameters and ease in GW quality monitoring. Furthermore, it contributes to solutions in data gaps when processed data are necessary.

The application of a range of machine learning techniques, as well as the combination and comparison of these approaches, resulted in a larger pool of potential environmental monitoring systems. The integration of machine learning techniques to spatial interpolation techniques have been implemented in several studies including Least Squares Support Vector Machine (LSSVM) and Population-based Incremental Learning to Ordinary Kriging (OK) [105], Random Forest (RF) to IDW and OK [106], Deep Neural Network (DNN) to Ordinary Kriging (OK) [107], Decision Tree to Kriging and Inverse Distance Weighting [108], and Non-linear AutoRegressive eXogenous (NARX) model to Geographic Information System [109].

A different method was integrated relative to spatial interpolation techniques in this research study. The use of the NN-PSO methodology with the capability of both prediction and optimization was utilized to enhance the prediction capacity of spatial interpolation methods. The findings of the cross validation of the HM concentration and physicochemical parameters showed that the spatial interpolation methods integrated with NN-PSO were the best method as manifested to its lowest MAE and highest R value. The governing interpolation methods were mostly under the geostatistical methods which integrated with NN-PSO including OK (OK+NN-PSO) and EBK (EBK+NN-PSO) method. Moreover, some HMs such as Ni (dry season) and Zn (wet season) have best performance using NN-PSO informed radial basis functions (RBF-NN-PSO). The findings of this study in reference to the performance of the NN-PSO informed spatial interpolation techniques agreed with the study of Li et al. [110], wherein they confirm that the integration of machine learning techniques produces more superior performance in the spatial interpolation method than compared to spatial interpolation methods without machine learning integration. The NN-PSO integration relative to spatial integration techniques addressed the issue in sampling density and was able to improve the performance of the spatial interpolation methods.

## 5. Conclusions

The objective of this research is to improve the mapping prediction capability of spatial interpolation algorithms by using an NN-PSO technique. It analyzed interpolation methods and mapping for physicochemical parameters such as temperature, pH, TDS, and EC, as well as mapping of HM concentrations in GW. This technique comprises three spatial interpolation methods such as deterministic, geostatistical, and interpolation with barriers with neuro-particle swarm optimization guided interpolation approaches. The measurement criteria for the best method were the least MAE and the highest R value.

The results recorded the governing interpolation techniques during the dry and wet season. The OK+NN-PSO method was recorded as best performing for temperature and EC during dry season, while EBK+NN-PSO for pH and TDS. The best methods during wet season were OK+NN-PSO for temperature and pH and EBK+NN-PSO for EC and TDS. These methods have the highest R and lowest MAE among the spatial interpolation techniques observed. The best methods for mapping physico-chemical characteristics were found to have the least MAE and R values ranging from 1.7 to 5.6 times higher than the interpolation techniques without NN-PSO integration.

The HM concentration maps during the dry season were observed to have the best performance using EBK+NN-PSO for Cr, Cd, Fe, Pb, Zn, and Cu while RBF+NN-PSO was the best method for Ni mapping. The best method during the wet season was found to be OK+NN-PSO for Cr, Cd, and Cu; EBK+NN-PSO for Fe, Mn, Ni, and Pb; and RBF+NN-PSO for Zn. The best method for mapping the GW HM concentrations during the wet season was observed to have the least MAE and R values ranging from 1.2 times to 5.6 times greater than the best interpolation method without NN-PSO integration.

Hybrid methods in general showed better performance than compared to the non-hybrid methods. The development of these hybrid methods using NN-PSO and geo-statistics provides a promising innovative approach for environmental quality monitoring as it improves the accuracy of predictive mapping and modelling of GW quality in an area. The integration of NN-PSO into spatial interpolation methods addresses the challenge of sample density and its effect on the spatial interpolation method’s performance. It opens a new avenue for enhancing the predictive capability of spatial interpolation algorithms. On the basis of the results of this research, it can be stated that the employment of models such as NN-PSO is suitable for overcoming the challenges in spatial data processing and mapping, as well as for improving the model’s predictive capabilities. The findings of the study suggest that the integration of ML techniques such as NN can be utilized in mapping GW quality as well as its application in spatio-temporal maps.

## Figures and Tables

**Table 1:** List of Watershed in Marinduque Province.

Watershed No.	Name of Watershed	Watershed No.	Name of Watershed
1	Hinanggayon—Mogpog	18	Catangon—Buenavista
2	Guisan—Mogpog	19	Libas—Buenavista
3	Balanacan—Mogpog	20	Lipata—Buenavista
4	Capayang—Mogpog	21	Buenavista
5	Laon—Mogpog	22	Dampulan—Torrijos
6	Sayao—Mogpog	23	Marlanga—Torrijos
7	Mogpog	24	Cabuyo—Torrijos
8	Pili—Boac	25	Matuyatuya—Torrijos
9	Murallon—Boac	26	Torrijos
10	Ihatub—Boac	27	Tambangan—Santa Cruz
11	Caganhao—Boac	28	Tawiran—Tagum
12	Maybo—Boac	29	Tagum—Santa Cruz
13	Bunganay—Boac	30	Botilao—Santa Cruz
14	Boac	31	Dolores—Santa Cruz
15	Banot—Gasan	32	Kamandugan—Santa Cruz
16	Dawis—Gasan	33	Hupi—Santa Cruz
17	Gasan	34	Santa Cruz

**Table 2:** Coordinates of the Sampling Locations.

Sampling Location Code	Barangay	Municipality	Latitude	Longitude	Elevation
BGW1	Tagwak	Boac	13.44552° N	121.87620° E	96 m
BGW2	Maligaya	Boac	13.47936° N	121.84087° E	10 m
BGW3	Puting Buhangin	Boac	13.45117° N	121.96087° E	282 m
BGW4	Balarin	Boac	13.41933° N	121.82200° E	17 m
BGW5	Bantay	Boac	13.43247° N	121.90953° E	208 m
BGW6	Hinapulan	Boac	13.41442° N	121.94785° E	242 m
BGW7	Boton	Boac	13.44292° N	121.86732° E	61 m
MGW1	Sumangga	Mogpog	13.47268° N	121.87412° E	68 m
MGW2	Nangka Dos (Site 1)	Mogpog	13.47972° N	121.85047° E	24 m
MGW3	Nangka Dos (Site 2)	Mogpog	13.47973° N	121.85053° E	24 m
MGW4	Janagdong	Mogpog	13.46952° N	121.85326° E	29 m
MGW5	Butansapa	Mogpog	13.48100° N	121.91803° E	145 m
MGW6	Putting Buhangin	Mogpog	13.45533° N	121.95198° E	265 m
BVGW1	Malbog (Site 1)	Buenavista	13.25813° N	121.94488° E	77 m
BVGW2	Malbog (Site 2)	Buenavista	13.26675° N	121.91648° E	103 m
BVGW3	Libas (Site 1)	Buenavista	13.25553° N	121.93958° E	69 m
BVGW4	Libas (Site 2)	Buenavista	13.26807° N	121.95612° E	70 m
BVGW5	Bagtingon	Buenavista	13.20521° N	121.99482° E	85 m
BVGW6	Sihi	Buenavista	13.25813° N	121.94488° E	371 m
GGW1	Banuyo	Gasan	13.27573° N	121.89303° E	5 m
GGW2	Masiga	Gasan	13.35505° N	121.82912° E	16 m
GGW3	Libtangin	Gasan	13.34647° N	121.83297° E	21 m
GGW4	Matandang Gasan	Gasan	13.32178° N	121.85268° E	46 m
GGW5	Dawis	Gasan	13.28638° N	121.88908° E	42 m
GGW6	Tiguion	Gasan	13.34365° N	121.86365° E	86 m
TGW1	Marlangga	Torrijos	13.32683° N	122.08442° E	56 m
TGW2	Poctoy (Site 1)	Torrijos	13.32943° N	122.09528° E	37 m
TGW3	Dampulan	Torrijos	13.22590° N	122.04562° E	25 m
TGW4	Sibuyao	Torrijos	13.34091° N	122.01261° E	444 m
TGW5	Poctoy (Site 2)	Torrijos	13.33164° N	122.01261° E	34 m
TGW6	Matuyatuya	Torrijos	13.37778° N	122.11611° E	15 m
SGW1	San Antonio	Santa Cruz	13.44612° N	121.98055° E	272 m
SGW2	Dolores (Site 1)	Santa Cruz	13.49177° N	121.96383° E	185 m
SGW3	Dolores (Site 2)	Santa Cruz	13.49183° N	121.96087° E	191 m
SGW4	Napo	Santa Cruz	13.43878° N	122.07607° E	65 m
SGW5	Matalaba	Santa Cruz	13.46595° N	122.05897° E	53 m

**Table 3:** Descriptive statistics for dry season physicochemical properties and HM concentrations in GW in the study area.

Parameter	Mean	PNSDW 2017 Guideline Value	WHO Guideline Value	Skewness	Kurtosis	CV%
Temp (°C)	36.80	-	-	0.417	−1.513	24.20
pH	7.02	6.5–8.5	6.5–9.2	−0.089	−1.678	10.30
EC (µS/cm)	935.17	-	1500	0.625	−1.166	88.40
TDS (mg/L)	372.77	600	1200	1.189	2.579	43.10
Cr (ppm)	0.06285	0.050	0.050	0.693	0.232	47.40
Cd (ppm)	0.03283	0.003	0.003	0.800	−1.300	140.16
Fe (ppm)	2.92944	1.000	0.300	4.026	14.917	378.74
Mn (ppm)	0.71753	0.400	0.400	3.165	9.205	264.87
Ni (ppm)	0.03902	0.070	0.070	0.754	−1.170	124.54
Pb (ppm)	0.05572	0.010	0.010	0.226	−1.779	94.68
Zn (ppm)	4.32901	5.000	3.000	3.374	10.135	299.87
Cu (ppm)	0.12688	1.000	2.000	0.212	−1.530	77.03

**Table 4:** Descriptive statistics for wet season physicochemical properties and HM concentrations in GW in the study area.

Parameter	Mean	PNSDW 2017 Guideline Value	WHO Guideline Value	Skewness	Kurtosis	CV%
Temp (°C)	31.55	-	-	0.800	−0.718	18.25
pH	7.43	6.5–8.5	6.5–9.2	0.474	−1.104	15.62
EC (µS/cm)	780.61	-	1500	1.082	−0.183	107.93
TDS (mg/L)	428.09	600	1200	0.978	−0.608	109.80
Cr (ppm)	0.08929	0.050	0.050	−0.048	−1.787	81.20
Cd (ppm)	0.06860	0.003	0.003	−0.695	−1.377	65.30
Fe (ppm)	16.0672	1.000	0.300	0.899	−1.095	143.96
Mn (ppm)	3.99553	0.400	0.400	0.186	−1.860	99.20
Ni (ppm)	0.05355	0.070	0.070	0.276	−1.749	100.63
Pb (ppm)	0.06298	0.010	0.010	−0.086	−1.904	89.07
Zn (ppm)	23.7530	5.000	3.000	0.358	−1.629	100.99
Cu (ppm)	0.13846	1.000	2.000	−0.103	−1.786	80.75

**Table 5:** Correlation analysis of HMs in GW during dry season.

	Temp	pH	EC	TDS	Cr	Cd	Fe	Mn	Ni	Pb	Zn	Cu
Temp	1.00	−0.20 **	0.24 **	−0.18 **	−0.06	−0.21 **	−0.19 **	−0.09	0.003	−0.11 *	−0.04	0.13 *
pH		1.00	0.06	−0.15 **	−0.07	−0.09	−0.12 *	0.18 **	−0.17 **	−0.18 **	0.03	−0.08
EC			1.00	0.30 **	−0.05	−0.28 **	0.05	0.12 *	−0.18 **	−0.23 **	−0.04	0.10
TDS				1.00	0.04	0.003	0.11 *	0.08	−0.17 **	−0.01	0.09	−0.20 **
Cr					1.00	0.72 **	0.01	0.001	0.69 **	0.81 **	0.09	0.33 **
Cd						1.00	−0.13 *	0.05	0.78 **	0.83 **	−0.11 *	0.27 **
Fe							1.00	0.12 *	−0.12 *	−0.04	0.50 **	−0.07
Mn								1.00	0.07	0.02	0.14 **	−0.09
Ni									1.00	0.77 **	−0.09	0.38 **
Pb										1.00	−0.03	0.41 **
Zn											1.00	−0.08
Cu												1.00

** Correlation is significant at the 0.01 level (two-tailed); * Correlation is significant at the 0.05 level (two-tailed).

**Table 6:** Correlation analysis of HMs in GW during wet season.

	Temp	pH	EC	TDS	Cr	Cd	Fe	Mn	Ni	Pb	Zn	Cu
Temp	1.00	0.04	−0.22 **	−0.22 **	0.01	0.11 *	−0.40 **	0.22 **	−0.09	−0.17 **	−0.07	0.03
pH		1.00	−0.11 *	−0.06	0.23 **	0.15 **	−0.06	0.36 **	−0.36 **	−0.28 **	0.33 **	0.49 **
EC			1.00	0.49 **	−0.30 **	−0.10	0.07	−0.52 **	−0.15 **	0.25 **	0.17 **	0.03
TDS				1.00	0.04	0.18 **	0.06	−0.19 **	0.18 **	0.04	−0.07	−0.08
Cr					1.00	0.40 **	−0.02	0.24 **	0.17 **	0.07	0.02	0.12 *
Cd						1.00	0.09	0.19 **	0.26 **	0.25 **	0.19 **	0.27 **
Fe							1.00	−0.08	−0.01	0.17 **	0.22 **	0.04
Mn								1.00	−0.24 **	−0.36 **	−0.12 *	0.05
Ni									1.00	0.19 **	−0.29 **	−0.21 **
Pb										1.00	0.05	−0.07
Zn											1.00	0.49 **
Cu												1.00

** Correlation is significant at the 0.01 level (two-tailed); * Correlation is significant at the 0.05 level (two-tailed).

**Table 7:** NN-PSO simulation results for GW quality parameters during the dry season.

	Hidden Neurons	No. of Particles	No. of Iterations	Elapsed Time (sec)	MSE	R	
						Validation	Testing
Temp	22	7	2000	214.984	0.11275	0.99988	0.99925
pH	27	5	2000	253.880	0.01176	0.98051	0.95432
EC	24	5	2000	215.542	0.03365	0.99993	0.99994
TDS	28	7	2000	220.718	0.00985	0.99061	0.99917
Cr	28	8	2000	147.899	0.00032	0.99958	0.98867
Cd	20	8	2000	110.308	0.00031	0.99669	0.98496
Fe	29	10	2000	142.590	0.01073	0.99683	0.99737
Mn	26	9	2000	146.913	0.00255	0.99788	0.99620
Ni	22	1	2000	115.371	0.00050	0.99987	0.99995
Pb	20	4	2000	115.961	0.00058	0.99949	0.99992
Zn	27	7	2000	110.420	0.00087	0.99972	0.99967
Cu	21	6	2000	138.813	0.00153	0.99985	0.99960

**Table 8:** NN-PSO simulation results for GW quality parameters during the wet season.

	Hidden Neurons	No. of Particles	No. of Iterations	Elapsed Time (s)	MSE	R	
						Validation	Testing
Temp	23	6	2000	226.663	0.73235	0.99440	0.99161
pH	23	6	2000	218.558	0.04694	0.99396	0.97383
EC	21	9	2000	223.412	0.02622	0.99755	0.99990
TDS	26	1	2000	205.010	0.00672	0.99360	0.99434
Cr	25	9	2000	147.159	0.00153	0.99948	0.99912
Cd	28	8	2000	145.022	0.00088	0.99998	0.99980
Fe	29	7	2000	147.002	0.18100	0.99992	0.99999
Mn	24	7	2000	157.724	0.01737	0.99938	0.99985
Ni	30	6	2000	155.655	0.15711	0.99498	0.98943
Pb	25	4	2000	109.265	0.00177	0.99998	0.98257
Zn	29	8	2000	178.172	0.17360	0.99305	0.99238
Cu	27	4	2000	174.482	0.00494	0.99882	0.99937

**Table 9:** Summary of the cross-validation performance of the models for the physicochemical parameters and the HM concentrations both for dry and wet season.

Parameter	Season	Governing Interpolation Method	MAE	R
Temperature	Dry	OK+NN-PSO	0.002000	0.941
pH	Dry	EBK+NN-PSO	0.001000	0.945
EC	Dry	OK+NN-PSO	0.002000	0.974
TDS	Dry	EBK+NN-PSO	0.001000	0.901
Cr	Dry	EBK+NN-PSO	0.000070	0.971
Cd	Dry	EBK+NN-PSO	0.000065	0.981
Fe	Dry	EBK+NN-PSO	0.045000	0.940
Mn	Dry	OK+NN-PSO	0.005000	0.922
Ni	Dry	RBF+NN-PSO	0.000200	0.991
Pb	Dry	EBK+NN-PSO	0.000100	0.989
Zn	Dry	EBK+NN-PSO	0.017000	0.951
Cu	Dry	EBK+NN-PSO	0.000200	0.974
Temperature	Wet	OK+NN-PSO	0.004000	0.925
pH	Wet	OK+NN-PSO	0.000300	0.976
EC	Wet	EBK+NN-PSO	0.000200	0.962
TDS	Wet	EBK+NN-PSO	0.001000	0.964
Cr	Wet	OK+NN-PSO	0.000100	0.963
Cd	Wet	OK+NN-PSO	0.000100	0.961
Fe	Wet	EBK+NN-PSO	0.055000	0.952
Mn	Wet	EBK+NN-PSO	0.005000	0.935
Ni	Wet	EBK+NN-PSO	0.000050	0.954
Pb	Wet	EBK+NN-PSO	0.000200	0.900
Zn	Wet	RBF+NN-PSO	0.019000	0.956
Cu	Wet	OK+NN-PSO	0.000200	0.926

## Data Availability

All data are contained in the manuscript.

## Appendix B

**Table A1:** Performance of methods used to interpolate GW quality parameters during dry season.

Parameter	Cross Validation	Deterministic Methods				Geostatistical Methods			Interpolation with Barriers	
		IDW	GPI	RBF	LPI	OK	UK	EBK	KS	DK
Temp *	MAE R	0.239 0.142	0.003 0.0003	0.006 0.082	0.059 0.092	0.038 0.055	0.086 0.071	0.066 0.004	0.089 0.183	0.081 0.004
Temp **	MAE R	0.030 0.889	0.009 0.112	0.006 0.916	0.057 0.933	0.002 0.941	0.026 0.939	0.029 0.940	0.060 0.934	0.099 0.757
pH *	MAE R	0.019 0.150	0.002 0.003	0.004 0.008	0.007 0.022	0.011 0.101	0.009 0.098	0.015 0.155	0.020 0.365	0.010 0.095
pH **	MAE R	0.003 0.905	0.010 0.108	0.002 0.920	0.004 0.930	0.004 0.943	0.009 0.942	0.001 0.945	0.001 0.938	0.002 0.796
EC *	MAE R	0.013 0.313	0.005 0.306	0.024 0.177	0.048 0.110	0.031 0.128	0.022 0.388	0.023 0.157	0.086 0.323	0.027 0.076
EC **	MAE R	0.003 0.940	0.010 0.278	0.002 0.952	0.004 0.964	0.002 0.974	0.018 0.970	0.004 0.973	0.007 0.970	0.015 0.849
TDS *	MAE R	0.002 0.111	0.002 0.545	0.005 0.001	0.023 0.039	0.019 0.158	0.022 0.154	0.013 0.182	0.028 0.195	0.008 0.080
TDS **	MAE R	0.004 0.863	0.003 0.155	0.003 0.869	0.006 0.887	0.002 0.887	0.003 0.890	0.001 0.901	0.004 0.888	0.006 0.861
Cr *	MAE R	0.0007 0.700	0.00008 0.605	0.0001 0.704	0.0007 0.679	0.0006 0.716	0.008 0.510	0.00007 0.683	0.0003 0.615	0.002 0.681
Cr **	MAE R	0.0002 0.966	0.0002 0.679	0.00008 0.968	0.0001 0.970	0.0001 0.967	0.002 0.957	0.00007 0.971	0.00007 0.970	0.0002 0.946
Cd *	MAE R	0.0006 0.822	0.00002 0.705	0.0002 0.819	0.0002 0.738	0.0005 0.832	0.002 0.552	0.0001 0.786	0.0003 0.738	0.003 0.713
Cd **	MAE R	0.0001 0.965	9.1 × 10^−5^ 0.699	0.0002 0.980	8.4 × 10^−5^ 0.977	0.00004 0.979	0.008 0.937	6.5 × 10^−5^ 0.981	1.5 × 10^−5^ 0.979	0.0001 0.898
Fe *	MAE R	0.269 0.077	0.020 0.169	0.090 0.134	0.283 0.089	0.375 0.095	0.167 0.039	0.038 0.160	0.120 0.010	0.078 0.160
Fe **	MAE R	0.187 0.906	0.500 0.258	0.068 0.906	0.135 0.920	0.046 0.932	0.540 0.739	0.045 0.940	0.101 0.920	0.100 0.742
Mn *	MAE R	0.105 0.127	0.006 0.199	0.050 0.041	0.011 0.195	0.029 0.185	0.086 0.125	0.022 0.228	0.040 0.155	0.031 0.294
Mn **	MAE R	0.027 0.841	0.006 0.089	0.006 0.857	0.007 0.879	0.005 0.922	0.841 0.815	0.007 0.908	0.008 0.889	0.019 0.645
Ni *	MAE R	0.0007 0.817	0.0003 0.707	0.0004 0.820	0.0003 0.737	0.0006 0.829	0.002 0.570	0.0006 0.780	0.0004 0.730	0.004 0.714
Ni **	MAE R	0.0003 0.963	0.004 0.666	0.0002 0.991	0.0003 0.979	0.0004 0.987	0.007 0.982	0.0003 0.986	0.0002 0.978	0.0005 0.883
Pb *	MAE R	0.0008 0.778	0.0005 0.673	0.0002 0.774	0.0009 0.710	0.0003 0.813	0.0010 0.632	0.0004 0.744	0.0003 0.702	0.003 0.691
Pb **	MAE R	0.0005 0.971	0.0001 0.740	0.0006 0.982	0.0003 0.985	0.0001 0.985	0.006 0.976	0.0001 0.989	0.0003 0.986	0.0008 0.906
Zn *	MAE R	0.130 0.177	0.054 0.325	0.310 0.247	0.149 0.067	0.993 0.082	0.121 0.187	0.183 0.395	0.669 0.125	0.111 0.428
Zn **	MAE R	0.449 0.908	0.018 0.363	0.157 0.927	0.350 0.938	0.073 0.946	0.074 0.879	0.017 0.951	0.255 0.926	0.381 0.739
Cu *	MAE R	0.0003 0.223	0.0004 0.246	0.003 0.212	0.0006 0.193	0.0005 0.251	0.073 0.335	0.002 0.189	0.0005 0.336	0.003 0.039
Cu **	MAE R	0.002 0.941	0.001 0.347	0.0008 0.953	0.0008 0.966	0.0005 0.972	0.007 0.961	0.0002 0.974	0.0004 0.965	0.003 0.850

** Neuro-particle swarm optimization informed; * Without neuro-particle swarm optimization.

**Table A2:** Performance of methods used to interpolate GW quality parameters during wet season.

Parameter	Cross Validation	Deterministic Methods				Geostatistical Methods			Interpolation with Barriers	
		IDW	GPI	RBF	LPI	OK	UK	EBK	KS	DK
Temp *	MAE R	0.166 0.050	0.011 0.039	0.037 0.053	0.067 0.083	0.060 0.111	0.035 0.118	0.146 0.107	0.333 0.118	0.084 0.236
Temp **	MAE R	0.060 0.890	0.010 0.311	0.026 0.923	0.046 0.902	0.004 0.925	0.010 0.916	0.005 0.922	0.019 0.911	0.088 0.780
pH *	MAE R	0.019 0.359	0.002 0.239	0.003 0.321	0.032 0.107	0.030 0.242	0.023 0.264	0.004 0.033	0.065 0.298	0.007 0.150
pH **	MAE R	0.011 0.916	0.0003 0.046	0.005 0.959	0.004 0.960	0.0003 0.976	0.0004 0.975	0.004 0.974	0.008 0.957	0.010 0.762
EC *	MAE R	0.007 0.030	0.001 0.224	0.003 0.026	0.032 0.008	0.002 0.127	0.026 0.104	0.003 0.048	0.077 0.030	0.007 0.073
EC **	MAE R	0.009 0.905	0.0005 0.309	0.002 0.934	0.002 0.945	0.0004 0.955	0.012 0.945	0.0002 0.962	0.005 0.953	0.0006 0.766
TDS *	MAE R	0.013 0.003	0.003 0.284	0.011 0.083	0.019 0.212	0.015 0.189	0.011 0.057	0.001 0.167	0.017 0.106	0.015 0.020
TDS **	MAE R	0.005 0.928	0.020 0.254	0.002 0.944	0.002 0.954	0.002 0.964	0.014 0.939	0.001 0.964	0.001 0.951	0.007 0.830
Cr *	MAE R	0.0008 0.206	0.0003 0.097	0.001 0.007	0.001 0.057	0.0006 0.149	0.051 0.168	0.003 0.128	0.007 0.015	0.0006 0.041
Cr **	MAE R	0.0001 0.912	0.001 0.138	0.0005 0.938	0.0005 0.932	0.0001 0.963	0.174 0.856	0.0004 0.960	0.0002 0.944	0.0003 0.637
Cd *	MAE R	0.004 0.304	0.0002 0.291	0.0007 0.570	0.005 0.599	0.0005 0.573	0.022 0.784	0.003 0.128	0.007 0.015	0.0006 0.041
Cd**	MAE R	0.0002 0.920	0.0003 0.218	0.0002 0.949	0.0002 0.936	0.0001 0.961	0.011 0.836	0.0001 0.957	0.0005 0.939	0.0002 0.747
Fe *	MAE R	0.373 0.386	0.038 0.114	0.823 0.181	0.612 0.129	0.198 0.272	0.197 0.271	0.328 0.302	0.969 0.064	0.661 0.136
Fe **	MAE R	0.064 0.924	0.002 0.376	0.030 0.949	0.101 0.950	0.025 0.942	0.055 0.705	0.055 0.952	0.107 0.931	0.068 0.745
Mn *	MAE R	0.545 0.169	0.007 0.477	0.132 0.326	0.236 0.434	0.239 0.326	0.712 0.641	0.029 0.374	0.375 0.342	0.090 0.322
Mn **	MAE R	0.014 0.878	0.003 0.244	0.002 0.926	0.051 0.920	0.005 0.930	0.264 0.786	0.005 0.935	0.042 0.927	0.071 0.715
Ni *	MAE R	0.002 0.519	0.0002 0.662	0.0001 0.622	0.003 0.488	0.00002 0.580	0.069 0.497	0.0009 0.531	0.0005 0.468	0.0009 0.125
Ni **	MAE R	0.0005 0.912	0.00004 0.252	0.0001 0.945	0.0005 0.938	0.00009 0.944	0.0105 0.754	0.00005 0.954	0.0003 0.941	0.0006 0.750
Pb *	MAE R	0.006 0.068	0.0003 0.285	0.0006 0.157	0.003 0.314	0.006 0.210	0.029 0.363	0.0007 0.337	0.003 0.284	0.0003 0.378
Pb **	MAE R	0.0004 0.862	0.002 0.193	0.0003 0.888	0.0003 0.863	0.0003 0.892	0.156 0.733	0.0002 0.900	0.0003 0.869	0.0006 0.681
Zn *	MAE R	0.263 0.172	0.060 0.727	0.810 0.162	0.941 0.154	0.204 0.358	0.389 0.728	0.158 0.778	0.167 0.213	0.018 0.594
Zn **	MAE R	0.037 0.905	0.015 0.038	0.019 0.956	0.033 0.898	0.023 0.949	0.399 0.847	0.063 0.937	0.098 0.904	0.023 0.721
Cu *	MAE R	0.009 0.687	0.0009 0.072	0.004 0.624	0.003 0.169	0.004 0.259	0.150 0.465	0.007 0.388	0.007 0.233	0.006 0.340
Cu **	MAE R	0.0009 0.917	0.0005 0.049	0.0006 0.915	0.0004 0.886	0.0002 0.926	0.226 0.813	0.0006 0.924	0.002 0.863	0.0004 0.722

** Neuro-particle swarm optimization informed; * Without neuro-particle swarm optimization.
